# The efficacy and safety of definitive concurrent chemoradiotherapy for non‐operable esophageal cancer

**DOI:** 10.1002/cam4.3724

**Published:** 2021-01-20

**Authors:** Alexandra D. Dreyfuss, Andrew R. Barsky, E. Paul Wileyto, Jennifer R. Eads, John C. Kucharczuk, Noel N. Williams, Thomas B. Karasic, James M. Metz, Edgar Ben‐Josef, John P. Plastaras, Andrzej P. Wojcieszynski

**Affiliations:** ^1^ Department of Radiation Oncology Perelman School of Medicine University of Pennsylvania Philadelphia PA USA; ^2^ Department of Biostatistics, Epidemiology, and Informatics Perelman School of Medicine University of Pennsylvania Philadelphia PA USA; ^3^ Division of Hematology and Oncology Perelman School of Medicine University of Pennsylvania Philadelphia PA USA; ^4^ Department of Surgery Hospital of the University of Pennsylvania Philadelphia PA USA

**Keywords:** concurrent chemoradiation, esophageal cancer, non‐operable, radiation therapy

## Abstract

**Objective:**

To report outcomes and toxicity in patients who received definitive concurrent chemoradiation (DCCRT) for non‐operable esophageal cancer (EC) in the modern era, and to identify markers of overall and disease‐free survival (OS/DFS).

**Methods:**

We conducted a retrospective cohort study of patients with unresectable EC who received DCCRT at our institution between 1/2008 and 1/2019. Descriptive statistics were used to report disease‐control outcomes and CTCAE v4.0–5.0 toxicities. Univariable and multivariable Cox regression, and stepwise regression were used to identify associations with survival.

**Results:**

At a median follow‐up of 19.5 months, 130 patients with adenocarcinoma (AC) (62%) or squamous cell carcinoma (SCC) (38%) were evaluable (Stage II‐III: 92%). Patients received carboplatin/paclitaxel (75%) or fluorouracil‐based (25%) concurrent chemotherapy. Median total RT dose was 50.4 Gy (range, 44.7–71.4 Gy) delivered in 28 fractions (24–35). Locoregional and distant recurrence occurred in 30% and 35% of AC, and 24% and 33% of SCC, respectively. Median OS and DFS were 22.9 and 10.7 months in AC, and 25.7 and 20.2 months in SCC, respectively. On stepwise regression, tumor stage, feeding tube during DCCRT, and change in primary tumor PET/CT SUVmax were significantly associated with OS and DFS. Most severe toxicities were acute grade 4 hematologic cytopenia (6%) and radiation dermatitis (1%). Most common acute grade 3 toxicities were hematologic cytopenia (35%), dysphagia (23%), and anorexia (19%).

**Conclusions:**

Treatment of non‐operable EC with DCCRT has acceptable toxicity and can provide multi‐year disease control for some patients, even in AC. Continued follow‐up and investigation in large studies would be useful.

## Graphical abstract

Treatment of non‐operable esophageal cancer with definitive concurrent chemoradiation has acceptable toxicity and can provide multi‐year disease control for some patients, even in adenocarcinoma.

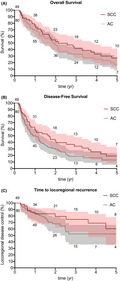

## 1 INTRODUCTION

Esophageal cancer (EC) is currently the eighth leading cause of cancer death, comprising 2–3% of all cancer deaths in the United States. In 2019, almost 18,000 new cases of EC were diagnosed, with a significant number initially presenting with locally advanced disease and only 20% surviving more than 5 years from time of diagnosis.^1^ Single modality therapy with surgery remains the mainstay of curative‐intent treatment for early stage cancers. For locally advanced disease, neoadjuvant chemoradiation (chemoRT) followed by esophagectomy is typically standard of care, demonstrating marked benefits in disease control and survival.^2^, ^3^, ^4^, ^5^, ^6^, ^7^, ^8^

In a subset of patients with locally advanced disease who are not candidates for or refuse surgery, definitive concurrent chemotherapy and radiation therapy (DCCRT) is the treatment of choice. To date, there have been several retrospective studies^9^, ^10^, ^11^ and few large prospective studies reporting on this approach. One randomized controlled trial compared outcomes in 172 patients with esophageal squamous cell carcinoma (SCC) treated with either induction chemotherapy followed by concurrent chemoradiotherapy (CCRT) and resection, or the same chemotherapy followed by CCRT with an additional 20.0 Gy of RT added instead of surgery. They found no difference in median survival time or 5‐ and 10‐year overall survival (OS) rates, with higher treatment‐related mortality observed in patients who received surgery.^12^ Similarly, the FFCD 9102 trial of 444 patients with potentially resectable T3N0‐1M0 esophageal SCC who received induction chemoRT followed by additional chemoRT or surgery found that while surgically treated patients had significantly lower rates of locoregional recurrence (LRR) (34% v 43%) and were less likely to require palliative intervention for dysphagia, they had similar 2‐year and median survival as compared to those who continued chemoRT, in addition to having worse quality of life outcomes and increased morbidity acutely.^13^ While these results are encouraging, neither trial included patients with adenocarcinoma (AC), which has grown to be the predominant EC in the United States.

As DCCRT continues to be used clinically, more data regarding outcomes and toxicities are needed, particularly for the use of more contemporary RT techniques and systemic therapies. This study reports our experience with DCCRT in the treatment of patients with non‐operable EC.

## 2 METHODS

### 2.1 Patient selection

We conducted a retrospective cohort study of patients who underwent DCCRT for non‐operable EC (by medical comorbidities, disease characteristics, or patient refusal of surgery) at our institution between 1/2008 and 1/2019. Inclusion criteria were histologically confirmed primary EC, T1a‐T4, N0‐N+ disease, Eastern Cooperative Group (ECOG) performance status <3, receipt of DCCRT to the esophagus with or without regional nodal RT, and at least one follow‐up visit with imaging (either with computed tomography (CT), positron emission tomography (PET)/CT or esophagogastroduodenoscopy (EGD) per the discretion of the treating physician) documented in the electronic medical record post DCCRT end date. In addition, as an RT dose of approximately 45 Gy is generally accepted as definitive, a dose cutoff of >44.5 Gy was implemented to ensure patients treated with palliative, neoadjuvant, or adjuvant RT doses were appropriately excluded. Patients met criteria for receiving DCCRT if at least 1 dose of chemotherapy was administered during the course of RT. Exclusion criteria were prior esophageal surgery, esophageal surgery <6 months post‐RT end date, or M+ disease.

Clinical staging information was extracted from the electronic medical record per the treating oncologist or radiologist notes and reflects American Joint Committee on Cancer (AJCC) seventh or eighth edition guidelines depending on time of diagnosis. Staging methods for the study population include CT chest/abdomen/pelvis, PET/CT, EGD, endoscopic ultrasound, and/or brain magnetic resonance imaging (MRI). The collection, storage, and retrieval of data were performed in compliance with the Institutional Review Board of The University of Pennsylvania and the Health Insurance Privacy and Portability Act.

The primary objectives were to report the efficacy and safety of DCCRT as determined by disease‐control outcomes and toxicity profiles. Secondary objectives were to identify characteristics associated with survival and recurrence. Toxicity grading was in accordance with the Common Terminology Criteria for Adverse Events (CTCAE) v4.0–5.0, depending upon year of DCCRT, using physician‐reported outcomes. Toxicity assessments were performed each week on treatment, 1–3 months after the end of treatment, and then every 6 months. Late toxicity was defined as >6 months post‐RT.

### 2.2 Treatment

#### 2.2.1 Radiotherapy

Patients underwent CT or PET/CT simulation in the supine position with a custom immobilization device. The gross tumor volume (GTV) was defined as the primary tumor and involved lymph nodes determined from CT, PET, clinical data, and endoscopic findings. The clinical target volume (CTV) was defined as the GTV plus a 3–4 cm cranio‐caudal expansion along the esophagus per the discretion of the treating radiation oncologist. Elective nodal volumes included the supraclavicular fossa for upper esophageal tumors and the celiac axis for lower tumors, at the discretion of the physician. An internal target volume (ITV) was generated to account for tumor motion due to normal breathing. The planning target volume (PTV) was defined as the ITV plus a 0.5 cm expansion. Patients were typically treated with 1.8 Gy daily fractions to a total initial dose of 45.0 Gy followed by a conedown of 5.4–14.4 Gy, based upon tumor location and physician discretion. All RT planning was done in Eclipse™ (Varian Medical Systems, Palo Alto, CA) (v11.0).

OAR dose constraints typically were lung V20 (volume receiving 20 Gy or more) <20%, V5<60%, mean <20 Gy, heart V40<50%, kidney V18<50%, spinal cord maximum 45 Gy, small bowel maximum 54 Gy, V50<5%, stomach mean <30 Gy, and mean liver <30 Gy. Deviations in target coverage and OAR doses were accepted at the discretion of the treating physician to ensure plan safety.

The RT modalities utilized included three‐dimensional conformal RT (3DCRT), intensity‐modulated RT (IMRT), and proton‐beam therapy (PBT). 3DCRT and fixed field IMRT beam arrangements were determined on a case‐by‐case basis to optimize target coverage and minimize dose to OARs. Volumetric modulated arc therapy (VMAT) was utilized instead of fixed field IMRT at the discretion of the treating physician. PBT was delivered either using double scatter, uniform scanning, or pencil‐beam scanning. PBT beam arrangements typically utilized cord‐sparing anterior fields or heart/lung‐sparing posterior fields, based upon the tumor location. Daily image‐guided RT was used.

#### 2.2.2 Systemic therapy

All patients received at least one dose of chemotherapy during the course of RT, with a minority of patients receiving induction and/or adjuvant/consolidation chemotherapy. Concurrent chemotherapy most commonly consisted of weekly carboplatin (AUC 2 mg/mL/min) and paclitaxel (50 mg/m^2^) for 5 weeks, beginning within one day of RT start and continuing until RT completion. Concurrent chemotherapy with cisplatin (60–100 mg/m^2^) and fluorouracil (750–1,000 mg/m^2^ continuous infusion 4–5 days per week) was administered as an alternative treatment regimen.

### 2.3 Statistics

Baseline and demographic characteristics were summarized using descriptive statistics. Cancer control and treatment‐related toxicity were reported using descriptive statistics, and Kaplan‐Meier analysis was used for OS and disease‐free survival (DFS). Differences in outcomes based on patient, disease, or treatment characteristics were tested for statistical significance with univariable (UVA) and multivariable (MVA) Cox regression, and stepwise regression. Variables were included in the MVA if *p* < 0.2 on UVA. For the stepwise regression, terms with *p* < 0.1 were eligible for addition to the model, and terms with *p* > 0.2 were eligible for removal. Fischer's exact test (for categorical variables) and Spearman's rank correlation (for ordinal variables) were used to assess patient, disease, and treatment associations with any type of recurrence, LRR, or distant metastasis (DM). Endpoints were calculated as time elapsed from date of diagnosis to date of death or last follow‐up. Graph Pad Prism (v8.1.2) was used to generate Kaplan‐Meier curves. Statistical analyses were conducted using Stata version 15 software (StataCorp, College Station, TX). All statistical tests considered *p* < 0.05 to represent statistical significance.

## 3 RESULTS

### 3.1 Patient and disease characteristics

A total of 130 patients (median age at diagnosis, 71; range, 44–92) were included in our analysis (Table 1). Males comprised a majority of the cohort (75%), as did Caucasians (80%), current or former smokers (81%), and AC tumor histology (62%). At initial presentation, 37% of patients had >10% weight loss over the prior 6 months. Summary stages were 5%, 29%, 46%, and 1% for stages I, II, III, and IV, respectively (stage I patients were not surgical candidates due to existing medical comorbidities or patient refusal of surgery, and the stage IV patient had T3N3M0 disease). The most common clinical tumor stages were T3 (61%) and T2 (18%), and 58% of patients presented with N+ disease.

**TABLE 1:** Patient and disease characteristics

Characteristic	AC (n = 80)	SCC (n = 49)	All^a^ (n = 130)
	No (%)		
Age at diagnosis (y)			
<60	14 (18)	8 (16)	23 (18)
60‐70	15 (19)	15 (31)	30 (23)
70‐80	31 (39)	21 (43)	52 (40)
>80	20 (25)	5 (10)	25 (19)
Sex			
Female	10 (12)	21 (43)	32 (25)
Male	70 (88)	28 (57)	98 (75)
Race			
Caucasian	73 (92)	30 (61)	104 (80)
African American	4 (5)	16 (33)	20 (16)
Other/Unknown	3 (5)	3 (6)	6 (4)
Smoker			
No	16 (20)	9 (18)	25 (19)
Yes	64 (80)	40 (82)	105 (81)
Barrett's esophagus			
No	53 (66)	47 (96)	101 (78)
Yes	27 (34)	2 (4)	29 (22)
PPI use pre‐diagnosis			
No	34 (43)	31 (63)	66 (51)
Yes	46 (58)	18 (38)	64 (49)
Weight loss, last 6 mo.			
10% body weight	49 (61)	33 (67)	82 (63)
>10% body weight	31 (39)	16 (33)	48 (37)
Tumor site, esophagus			
Upper third	0 (0)	6 (12)	7 (5)
Middle third	2 (3)	22 (45)	24 (18)
Lower third/GEJ	65 (81)	16 (33)	81 (62)
Unknown	13 (16)	5 (10)	18 (14)
Summary stage^b^			
I	6 (8)	1 (2)	7 (5)
II	25 (31)	13 (27)	38 (29)
III	36 (45)	24 (49)	60 (46)
IV	0 (0)	1 (2)	1 (1)
Unknown	13 (16)	10 (20)	24 (18)
Clinical tumor stage^b^			
T1	3 (4)	3 (6)	6 (5)
T2	17 (21)	7 (14)	24 (18)
T3	52 (65)	27 (55)	79 (61)
T4	2 (3)	5 (10)	7 (5)
Unknown	6 (8)	7 (14)	14 (11)
Clinical nodal stage^b^			
N0	27 (34)	21 (43)	48 (37)
N+	50 (63)	26 (53)	76 (58)
Unknown	3 (4)	2 (4)	6 (5)

Abbreviations: AC, adenocarcinoma; AJCC, American Joint Committee on Cancer; GEJ, gastroesophageal junction; PPI, proton pump inhibitor; SCC, squamous cell carcinoma.

^a^ Includes one unknown tumor histology

^b^ Stages reflect staging system at time of diagnosis, including both AJCC 7th and 8th editions.

### 3.2 Treatment characteristics

Details of chemotherapy regimens and RT are summarized in Table 2. Concurrent chemotherapy regimens included a median of 5 cycles (1–8) of carboplatin/paclitaxel (75%) or a median of 5 weeks (2–8) of continuous infusion fluorouracil‐based therapy (25%) given concurrently with RT. Chemotherapy dose reduction or treatment breaks were required in 35% and 48% of patients who received carboplatin/paclitaxel or fluorouracil‐based therapy, respectively, and the median percent of intended concurrent chemotherapy cycles received was 100.0% (20.0–100.0%) and 95.0% (50.0–100.0%), respectively. Most patients did not receive additional chemotherapy (60%), but 15% received induction chemotherapy, 23% received adjuvant/consolidation chemotherapy, and 2% received both. Half of patients had feeding tubes in place pre‐RT, with 7 requiring feeding tube placement during or within 4 weeks of RT completion.

**TABLE 2:** Details of chemotherapy and radiation therapy

Characteristic	AC (n = 80)	SCC (n = 49)	All (n = 130)
	No (% or range)		
Chemotherapy			
Concurrent regimen^a^			
Carboplatin/paclitaxel	58 (73)	39 (80)	97 (75)
Fluorouracil/‐platin	9 (11)	9 (18)	18 (14)
Fluorouracil only	11 (14)	0 (0)	11 (8)
Other^a^	2 (3)	1 (2)	4 (3)
Median % of concurrent regimen received			
Carboplatin/paclitaxel^b^	100.0 (20.0–100.0)	100.0 (20.0–100.0)	100.0 (20.0–100.0)
Fluorouracil‐based^a^	100.0 (66.7–100.0)	66.7 (50.0–100)	95.0 (50.0–100.0)
Chemotherapy dose reduction or break			
Carboplatin/paclitaxel	19 (32)	16 (40)	35 (35)
Fluorouracil‐based	8 (38)	7 (78)	15 (48)
Other chemotherapy			
None	50 (63)	27 (55)	78 (60)
Induction	14 (18)	5 (10)	19 (15)
Adjuvant/consolidation	16 (20)	14 (29)	30 (23)
Both	0 (0)	3 (6)	3 (2)
Feeding tube^c^			
No	46 (58)	19 (39)	65 (50)
Yes	34 (43)	30 (61)	65 (50)
Radiation therapy			
Modality			
3DCRT	13 (16)	7 (14)	20 (15)
IMRT/PBT	67 (84)	42 (86)	110 (85)
Median total dose (Gy)	50.4 (45.0–61.2)	54.0 (44.7–71.4)	50.4 (44.7–71.4)
Median total # fractions	28 (24–34)	30 (25–34)	28 (24–35)
Median initial dose (Gy)	45.0 (36.0–61.2)	45.0 (32.4–68.0)	45.0 (32.4–70.0)
Median initial # fractions	25 (20–34)	25 (18–34)	25 (18–35)
Conedown			
No	7 (9)	4 (8)	12 (9)
Yes	73 (91)	44 (92)	118 (91)
Median conedown dose (Gy)	5.4 (1.8–14.4)	9.0 (5.4–18.0)	5.4 (1.8–18.0)
Median # conedown fractions	3 (1–9)	5 (3–10)	3 (1–10)

Abbreviations: 3DCRT, 3‐D conformal radiation therapy; AC, adenocarcinoma; IMRT, intensity‐modulated radiation therapy; PBT, proton beam therapy; RT, radiation therapy; SCC, squamous cell carcinoma.

^a^ Pre‐2014, only 45% of the patients treated received chemotherapy with carboplatin/paclitaxel received. During and post‐2014, 92% of patients treated received chemotherapy with carboplatin/paclitaxel.

^b^ Paclitaxel (n = 1) and cisplatin/paclitaxel (n = 1) included in carboplatin/paclitaxel group, fluorouracil/cisplatin +epirubicin (n = 1), fluorouracil/cisplatin +cetuximab (n = 1) included in fluorouracil‐based group for subsequent analyses.

^c^ Feeding tube placement (n = 7) or removal (n = 1) required during or <4 weeks post‐RT end.

For the RT component of therapy, patients received either 3DCRT (15%), IMRT (52%), or PBT (32%). The median total dose was 50.4 Gy (44.7–71.4 Gy) delivered in a median of 28 fractions (24–35). A conedown was delivered in 91% of patients to a median dose of 5.4 Gy (1.8–18.0 Gy) in a median of 3 fractions (1–10).

### 3.3 Toxicity

The most severe acute and late toxicity outcomes are reported in Table 3 and Supplementary Table S1, respectively. Worst toxicities were 8 (6%) acute grade 4 hematologic cytopenias and 1 (1%) acute grade 4 radiation dermatitis, which occurred in a patient treated to a dose of 70.0 Gy for an upper esophageal tumor. Among the 99 carboplatin/paclitaxel and 25 fluorouracil‐based therapy patients with acute toxicity data, grade 3 toxicities involved the upper aerodigestive tract (37% vs 28%), gastrointestinal tract (24% vs 24%), hematologic cytopenia (37% vs 24%), skin (5% vs 0%), nervous system (1% vs 0%), and fatigue (6% vs 0%).

**TABLE 3:** Number of most severe acute (≤6 months post‐radiation therapy) grade 1–5 toxicities (%)

Toxicity grade	Carboplatin/paclitaxel (n = 99)				Fluorouracil‐based (n = 25)^a^			
	1	2	4‐Mar	Total	1	2	4‐Mar	Total
Upper aerodigestive tract								
Cough	28 (28)	4 (4)	0	32 (32)	2 (8)	0	0	2 (8)
Dyspnea	15 (15)	5 (5)	4 (4)	24 (24)	3 (12)	0	0	3 (12)
Dysphagia	14 (14)	36 (36)	26 (26)	76 (76)	3 (12)	6 (24)	3 (12)	12 (48)
Mucositis	5 (5)	1 (1)	1 (1)	7 (7)	1 (4)	2 (8)	3 (12)	6 (24)
Esophageal pain	35 (35)	21 (21)	1 (1)	57 (57)	6 (24)	2 (8)	0	8 (32)
Esophagitis	20 (20)	23 (23)	5 (5)	48 (48)	1 (4)	9 (36)	1 (4)	11 (44)
Hoarseness	25 (25)	0	0	25 (25)	1 (4)	0	0	1 (4)
Gastrointestinal								
Anorexia	24 (24)	34 (34)	21 (21)	79 (79)	2 (8)	4 (16)	3 (12)	9 (36)
Dehydration	29 (29)	13 (13	2 (2)	44 (44)	5 (20)	3 (12)	1 (4)	9 (36)
Nausea/vomiting	36 (36)	18 (18)	1 (1)	55 (55)	6 (24)	3 (12)	0	9 (36)
Diarrhea	24 (24)	2 (2)	0	26 (26)	5 (20)	3 (12)	2 (8)	10 (40)
Hematologic^b^								
Cytopenia	13 (13)	39 (39)	44 (44)^c^	96 (96)	7 (28)	11 (44)	7 (28)^d^	25 (100)
Skin								
Radiation dermatitis	38 (38)	8 (8)	5 (5)	51 (51)	7 (28)	2 (8)	1 (4)^e^	10 (40)
Other								
Fatigue	47 (47)	42 (42)	6 (6)	93 (93)	10 (40)	5 (20)	0	15 (60)
Depression	26 (26)	4 (4)	0	30 (30)	5 (20)	1 (4)	0	6 (24)
Neuropathy	5 (5)	5 (5)	1 (1)	11 (11)	6 (24)	4 (16)	0	10 (40)

^a^ Six patients did not have acute toxicity data available.

^b^ Includes anemia, thrombocytopenia, neutropenia, leukopenia.

^c^ 7 (7%) grade 4 hematologic cytopenia.

^d^ 1 (4%) grade 4 hematologic cytopenia.

^e^ 1 (4%) grade 4 radiation dermatitis.

### 3.4 Disease‐control outcomes

**FIGURE 1:**
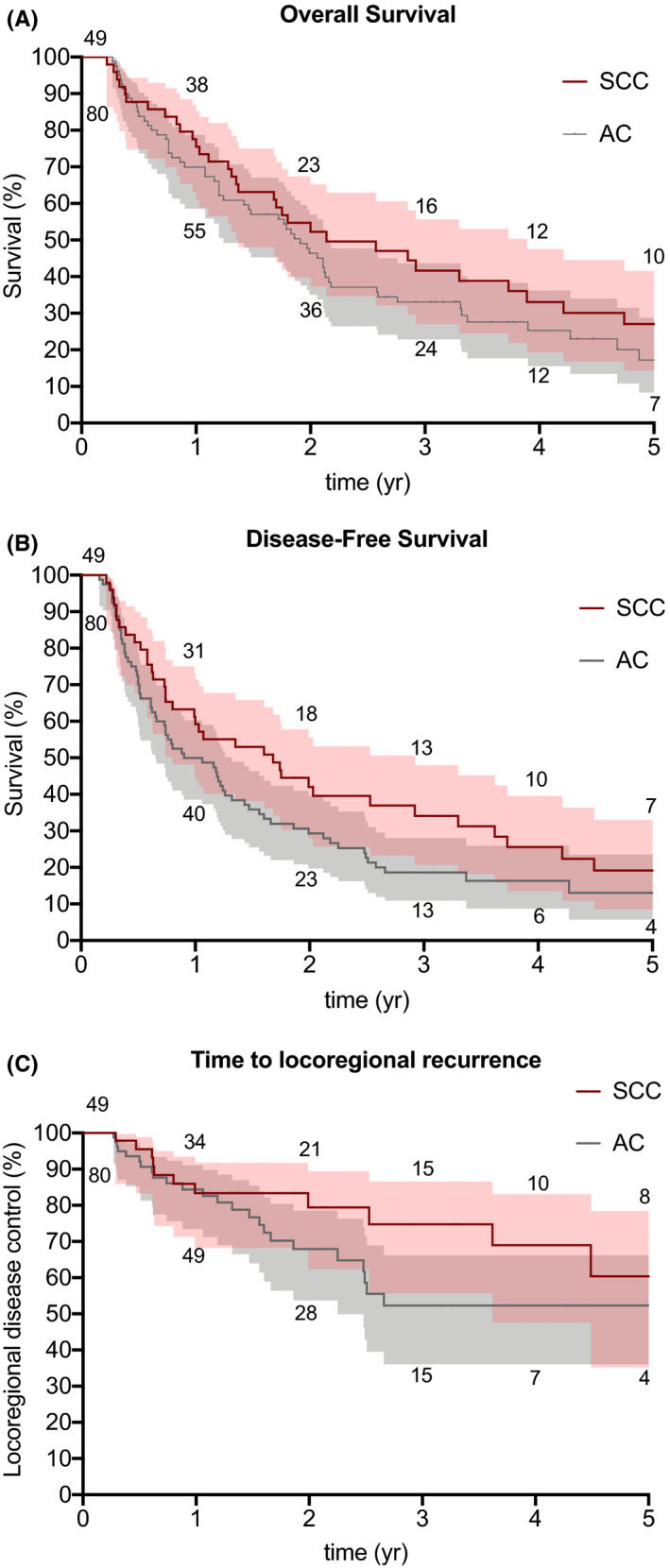
A, Kaplan‐Meier modeling of overall survival by tumor histology. B, Kaplan‐Meier modeling of disease‐free survival by tumor histology. C, Time to locoregional recurrence by tumor histology. Numbers indicate number of patients at risk and shading indicates 95% confidence intervals. SCC, squamous cell carcinoma; AC, adenocarcinoma

Disease outcome measures are summarized in Table 4, and Kaplan‐Meier analyses for OS, DFS, and time to LRR stratified by tumor histology are depicted in Figure 1. Median follow‐up time was 19.5 months (2.5–145.0), with 25% of patients alive at the time of data analysis, and 15 patients alive at 5 years post‐treatment (6 AC, 9 SCC). Median OS was 22.9 months [95% confidence interval (CI), 14.8, 25.9] in AC and 25.7 months [16.4, 46.7] in SCC. Median DFS was 10.7 months [7.8, 15.8] in AC and 20.2 [9.7, 35.1] in SCC. OS and DFS at 1 year were 69% [57, 78] for AC and 74% [59, 84] for SCC patients, respectively. No patients were considered lost to follow‐up, as all were evaluated at least once following treatment completion. In addition, all patients alive at their respective time of last follow‐up were evaluated within 1 year of time of data analysis, with the exception of four patients whose time to last follow‐up was still 22, 38, 68, and 77 months.

**TABLE 4:** Disease‐control outcomes

Characteristic	AC (n = 80)	SCC (n = 49)	All (n = 130)
	No; (%), (range), or [95% CI]		
Median follow‐up time (mo.)	17.2 (2.6–96.7)	21.0 (2.5–145.0)	19.5 (2.5–145.0)
Alive at analysis time			
No	59 (74)	38 (78)	98 (75)
Yes	21 (26)	11 (22)	32 (25)
Median overall survival (mo.)	22.9 [14.8, 25.9]	25.7 [16.4, 46.7]	23.6 [17.7, 26.2]
Carboplatin/paclitaxel	22.3 [14.4, 25.6]	34.2 [20.2, 56.9]	24.0 [17.7, 31.0]
Fluorouracil‐based	24.7 [9.1, 58.4]	16.4 [3.7, 46.7]	20.6 [12.4, 46.7]
Median disease‐free survival (mo.)	10.7 [7.8, 15.8]	20.2 [9.7, 35.1]	14.0 [9.1, 18.7]
LRR			
No	56 (70)	37 (76)	94 (72)
Yes	24 (30)	12 (24)	36 (28)
Median time to LRR (yr.)	NR [2.3, NR]	12.2 [3.6, NR]	12.2 [2.7, NR]
DM			
No	52 (65)	33 (67)	86 (66)
Yes	28 (35)	16 (33)	44 (34)
Median time to DM (yr.)	4.8 [1.9, NR]	NR [2.0, NR]	4.8 [2.5, NR]
LRR or DM			
No	40 (50)	28 (57)	69 (53)
Yes	40 (50)	21 (43)	61 (47)
Median time to LRR or DM (mo.)	19.2 [14.3, 30.1]	43.5 [20.8, NR]	27.0 [17.6, 43.5]
Site of DM^a^			
Liver	10 (36)	3 (19)	13 (30)
Bone	4 (14)	6 (38)	10 (23)
Lung	2 (7)	5 (31)	7 (16)
Brain	3 (11)	0 (0)	3 (7)
Combination	4 (14)	2 (13)	6 (14)
Other	5 (18)	0 (0)	5 (11)
Median % change in primary PET/CT SUVmax	61.7 (−40.5–100.0)	72.3 (−3.8–100.0)	69.6 (−40.5–100.0)
Change in ECOG performance score			
‐	4 (5)	6 (12)	10 (8)
0	34 (43)	21 (43)	56 (43)
+	34 (43)	13 (27)	47 (36)
Unknown	8 (10)	9 (18)	17 (13)

Abbreviations: AC, adenocarcinoma; DM, distant metastasis; ECOG, eastern cooperative group; LRR, locoregional carcinoma; NR, not reached; PET SUVmax, positron emission tomography / computed tomography maximum standardized uptake value; SCC, squamous cell carcinoma.

^a^ Combinations: Bone/liver (n = 3), bone/lung (n = 1), liver/lung (n = 1), bone/liver/lung (n = 1); Other: peritoneum (n = 2), paraspinal muscle (n = 1), bowel (n = 1), peritoneum/pleura (n = 1).

LRR occurred in 30% of AC and 24% of SCC patients, and DM occurred in 35% AC and 33% of SCC patients, with a median time to either recurrence of 19.2 [14.3, 30.1] and 43.5 [20.8, NR] in AC and SCC, respectively. The median percent change in primary PET/CT SUVmax pre‐ and post‐RT was 61.7% (−40.5–100.0) in AC and 72.3% (−3.8–100.0%) in SCC. ECOG performance status worsened following RT in 36% of patients.

Table 5 shows the results of the UVA and MVA Cox regression, and stepwise regression for OS. Variables with *p*‐value<0.2 on UVA were included in the MVA, which showed T3 disease (*p* = 0.038), RT dose (*p* = 0.018), and change in primary tumor PET/CT SUVmax (*p* = 0.032) to be significantly associated with OS. (T4 disease trended towards significance (*p* = 0.056)). On stepwise regression, increased clinical tumor stage (*p* = 0.013), no feeding tube post‐chemoRT (<0.001), RT dose (*p* = 0.008), and <50% change in primary tumor PET/CT SUVmax (*p* = 0.005) were significantly associated with worse OS.

**TABLE 5:** Univariable and multivariable Cox regression, and stepwise regression of overall survival

Variable		n			Mean OS ± SD (mo.)		UVA				MVA				Stepwise			
							HR [95% CI]		*p*‐value		HR [95% CI]		*p*‐value		HR [95% CI]		*p*‐value	
Age at diagnosis (yr)																		
<60	23			27.8 ± 33.4			‐		0.718						‐			
60–70	30			26.3 ± 23.5			0.8 [0.5, 1.6]											
70–80	52			31.9 ± 25.4			0.7 [0.4, 1.3]											
>80	25			27.5 ± 22.5			0.7 [0.4, 1.4]											
Sex																		
Female	32			31.8 ± 26.8			‐		**0.154**		**‐**		0.953		‐			
Male	98			28.2 ± 25.7			1.4 [0.9, 2.3]				1.0 [0.4, 2.2]							
Smoker																		
No	25			28.0 ± 25.4			‐		0.812						‐			
Yes	105			29.3 ± 26.2			0.9 [0.6, 1.5]											
Barrett's esophagus																		
No	101			27.7 ± 24.9			‐		0.364						‐			
Yes	29			34.0 ± 29.1			0.8 [0.5, 1.3]											
PPI use pre‐diagnosis																		
No	66			30.1 ± 29.4			‐		0.445						‐				
Yes	64			28.1 ± 22.0			0.9 [0.6, 1.3]												
Body weight loss, last 6 mo.																		
≤10%	82			34.3 ± 28.0			‐		**0.003**		**‐**		0.879		‐			
>10%	48			20.1 ± 19.0			1.9 [1.2, 2.8]				1.1 [0.5, 2.1]							
Tumor histology																		
AC	80			26.9 ± 23.0			‐		0.532						1.2 [0.9, 1.4]		0.162	
SCC	49			33.1 ± 30.0			0.9 [0.6, 1.4]											
Tumor site, esophagus^a^																		
Upper	7			49.8 ± 35.1			‐								‐			
Middle	24			27.4 ± 21.1			1.5 [0.6, 3.8]		0.535									
Lower/GEJ	81			25.4 ± 21.9			1.6 [0.7, 3.7]											
Clinical tumor stage^a^																		
T1	6			57.8 ± 30.4			‐		**<0.001**		**‐**		‐		1.8 [1.1, 3.0]		**0.013**	
T2	24			32.4 ± 20.6			6.3 [0.8, 47.7]				4.6 [0.6, 37.1]		0.160					
T3	79			23.7 ± 20.5			11.3 [1.6, 82.2]				8.9 [1.1, 69.7]		**0.038**					
T4	7			18.0 ± 19.7			18.6 [2.3, 152.8]				9.2 [0.9, 89.1]		0.056					
Clinical nodal stage^a^																			
N0			48			27.7 ± 23.0		‐		0.428						1.9 [1.0, 3.7]		0.061	
N+			76			27.3 ± 22.9		1.2 [0.8, 1.8]											
Feeding tube pre‐chemoRT																			
No			65			36.5 ± 29.3		‐		**<0.001**		**‐**		0.548		‐			
Yes			65			21.6 ± 19.6		2.6 [1.7, 4.0]				1.5 [0.4, 45.9]							
Feeding tube post‐chemoRT																			
No			59			38.2 ± 30.3		‐		**<0.001**		**‐**		0.139		4.3 [2.0, 9.4]		**<0.001**	
Yes			71			21.5 ± 19.0		2.7 [1.8, 4.2]				2.9 [0.7, 12.2]							
Concurrent chemotherapy^a^																			
Carbo/tax			99			27.5 ± 23.8		‐		0.853						‐			
Flu‐based			31			34.0 ± 32.6		1.0 [0.6, 1.5]											
RT dose (Gy)																			
‐			‐			‐		0.9997 [0.9992, 1.0]		**0.194**		0.999 [0.998, 0.9998]		**0.018**		0.999 [0.998, 0.9997]		**0.008**	
RT modality																			
3DCRT			20			19.0 ± 19.0		‐		**0.004**		‐		0.819		**‐**			
IMRT/PBT			110			30.9 ± 26.7		0.5 [0.3, 1.8]				0.9 [0.3, 2.5]							
Change in primary tumor PET SUV^a^																			
<50%			22			25.5 ± 19.9		‐		**0.137**		‐		**0.032**		0.3 [0.2, 0.7]		**0.005**	
50–100%			52			36.9 ± 25.7		0.6 [0.4, 1.2]				0.5 [0.2, 0.9]							

Bold values are statistically significant.

Abbreviations: 3DCRT, 3‐D conformal radiation therapy; Carbo/tax, carboplatin/paclitaxel; chemoRT, chemoradiation; CI, confidence interval; Flu‐based, fluourouracil‐based; GEj, gastroesophageal junction; HR, hazard ratio; IMRT, intensity modulated radiation therapy; MVA, multivariable analysis; OS, overall survival; PBT, proton beam therapy; PET/CT SUVmax, positron emission tomography / computed tomography maximum standardized uptake valuePPI, proton pump inhibitor; RT, radiation therapy; SD, standard deviation; UVA, univariable analysis.

^a^ Unknown / other omitted for brevity.

Table 6 shows the corresponding analysis for DFS. Variables with *p*‐values <0.2 on UVA which were included in and significant on MVA were sex (*p* = 0.038) and T3 (*p* = 0.009) and T4 (*p* = 0.004) disease. Stepwise regression revealed significant associations between worse DFS and male sex (*p* = 0.036), increased clinical tumor stage (*p* = 0.001), no feeding tube pre‐chemoRT (*p* = 0.017), RT dose (*p* = 0.013), and <50% change in primary tumor PET/CT SUVmax (*p* = 0.016).

**TABLE 6:** Univariable and multivariable Cox regression, and stepwise regression of disease‐free survival

Variable		n		Mean DFS ± SD (y)	UVA		MVA		Stepwise	
					HR [95% CI]	*p*‐value	HR [95% CI]	*p*‐value	HR [95% CI]	*p*‐value
Age at diagnosis (y)										
<60	23		18.0 ± 30.4		‐	0.380			‐	
60–70	30		20.6 ± 22.5		0.7 [0.4, 1.2]					
70–80	52		22.3 ± 20.3		0.7 [0.4, 1.1]					
>80	25		24.1 ± 23.0		0.6 [0.3, 1.1]					
Sex										
Female	32		27.8 ± 26.6		‐	**0.028**	‐	**0.038**	2.3 [1.1, 4.8]	**0.036**
Male	98		19.4 ± 21.7		1.7 [1.1, 2.8]		2.5 [1.1, 6.1]			
Smoker										
No	25		19.7 ± 21.6		‐	0.666			‐	
Yes	105		21.9 ± 23.6		0.9 [0.6, 1.5]					
Barrett's esophagus										
No	101		21.9 ± 23.8		‐	0.632			‐	
Yes	29		20.1 ± 20.9		1.1 [0.7, 1.7]					
PPI use pre‐diagnosis										
No	66		22.0 ± 27.2		‐	0.292			‐	
Yes	64		20.9 ± 18.2		0.8 [0.6, 1.2]					
Body weight loss, last 6 mo.										
≤10%	82		24.1 ± 25.2		‐	**0.120**	‐	0.199	‐	
>10%	48		17.0 ± 18.5		1.3 [0.9, 2.0]		0.7 [0.4, 1.2]			
Tumor histology										
AC	80		18.1 ± 17.9		‐	**0.114**	‐	‐	‐	
SCC	49		27.3 ± 29.2		0.7 [0.5, 1.1]		0.9 [0.5, 1.7]	0.817		
Tumor site, esophagus^a^										
Upper	7		28.2 ± 36.3		‐					
Middle	24		23.2 ± 19.5		0.8 [0.3, 2.2]	0.443			‐	
Lower/GEJ	81		17.1 ± 16.6		1.2 [0.5, 2.7]					
Clinical tumor stage^a^										
T1	6		51.7 ± 36.1		‐	**0.001**	‐	‐	2.9 [1.6, 5.2]	**0.001**
T2	24		26.7 ± 21.1		3.0 [0.7, 13.2]		2.3 [0.5, 11.0]	0.310		
T3	79		15.3 ± 13.1		6.5 [1.5, 26.7]		8.6 [1.7, 42.7]	**0.009**		
T4	7		10.1 ± 14.9		13.2 [2.7, 65.1]		18.8 [2.6, 137.7]	**0.004**		
Clinical nodal stage^a^										
N0	48		21.6 ± 22.5		‐	0.387			‐	
N+	76		19.3 ± 18.5		1.2 [0.8, 1.8]					
Feeding tube pre‐chemoRT										
No	65		27.0 ± 26.9		‐	**0.001**	‐	0.129	2.5 [1.2, 5.2]	**0.017**
Yes	65		16.0 ± 17.3		2.1 [1.4, 3.1]		2.8 [0.7, 10.8]			
Feeding tube post‐chemoRT										
No	59		27.1 ± 27.9		‐	**0.002**	‐	0.296	‐	
Yes	71		16.8 ± 17.2		1.9 [1.3, 2.8]		0.5 [0.1, 1.8]			
Concurrent chemotherapy^a^										
Carbo/tax	99		21.9 ± 23.4		‐	0.			‐	
Flu‐based	31		20.2 ± 22.7		1.2 [0.8, 1.9]					
RT dose (Gy)										
‐	‐		‐		0.9998 [0.999, 1.0]	0.289			0.999 [0.998, 0.9998]	**0.013**
RT modality										
3DCRT	20		11.8 ± 14.8		‐	**0.002**	‐	0.318	‐	
IMRT/PBT	110		23.2 ± 24.0		0.5 [0.3, 0.8]		0.6 [0.3, 1.6]			
Change in primary tumor PET/CT SUVmax^a^										
<50%	22		17.9 ± 19.5		‐	**0.146**	‐	0.137	0.5 [0.2, 0.9]	**0.016**
50–100%	52		24.9 ± 23.7		0.7 [0.4, 1.1]		0.6 [0.3, 1.2]			

Bold values are statistically significant.

Abbreviations: 3DCRT, 3‐D conformal radiation therapy; Carbo/tax, carboplatin/paclitaxel; chemoRT, chemoradiation; CI, confidence interval; Flu‐based, fluourouracil‐based; GEj, gastroesophageal junction; HR, hazard ratio; IMRT, intensity modulated radiation therapy; MVA, multivariable analysis; OS, overall survival; PBT, proton beam therapy; PET/CT SUVmax, positron emission tomography / computed tomography maximum standardized uptake valuePPI, proton pump inhibitor; RT, radiation therapy; SD, standard deviation; UVA, univariable analysis.

^a^ Unknown / other omitted for brevity.

Lastly, we evaluated potential associations between patient, disease, and treatment characteristics and recurrence, with specific analyses for LRR and DM carried out separately. Results are reported in Supplementary Table S2.

## 4 DISCUSSION

For patients with EC, treatment can vary considerably and is dictated by disease stage, surgical candidacy, and patient preference. Current guidelines recommend endoscopic therapy for mucosal tumors and surgery for more invasive tumors. For cancers that have metastasized to the lymph nodes, neoadjuvant therapies are typically added to treatment plans as components of trimodality therapy.^5^, ^14^, ^15^ Given the significant morbidity and decreased quality of life associated with esophageal surgery in some circumstances, an organ preservation approach using DCCRT has made significant inroads in the treatment of EC.^16^ However, efficacy and safety data from large, modern experiences are still lacking, especially for patients with AC. Here we report our experience treating 130 patients with non‐operable AC or SCC of the esophagus with DCCRT.

In our current study, we observed acceptable short‐term efficacy with DCCRT in a considerable number of esophageal AC and SCC patients. In AC patients, median OS was 22.9 months and median DFS was 10.7 months. Patients with SCC had improved DFS and OS, at 25.7 and 20.2 months, respectively. Our results are consistent with previously published data on non‐operative approaches to EC, and reveal a noteworthy improvement in survival when compared to older data, perhaps in part due to advancements in RT delivery (84% IMRT/PBT), chemotherapy regimens, and the use of PET/CT in a majority (77%) of patients to inform treatment planning. Herskovic et al. reported a median OS of 12.5 months and 1‐year OS of 50% in 61 patients with T1‐T3 nonmetastatic EC (86% SCC) treated with four cycles of combined fluorouracil and cisplatin plus 50.0 Gy with 14.0 Gy conedown.^4^ In contrast to our study, all patients were treated with 2DCRT or 3DCRT. In a more recent multicenter, parallel‐group phase 2/3 trial of patients with stage I‐IVa EC treated with either six cycles (3 concomitant with 50.0 Gy RT in 25 fractions) of leucovorin, fluorouracil, and oxaliplatin (FOLFOX) or four cycles (2 concurrent with 50.0 Gy RT in 25 fractions) of fluorouracil and cisplatin, Conroy et al. reported median OS of 20.2 and 17.5 months, respectively, and median progression‐free survival of 9.7 and 9.4 months, respectively.^17^ Furthermore, in the few randomized trials that have directly compared operative to non‐operative therapy, no significant difference in median survival time or 2‐, 5‐, and 10‐year OS rates were observed, with higher treatment‐related mortality and acute morbidity in surgery patients, albeit with decreased rates of LRR (34% vs 43%).^12^, ^13^ Still, our observed LRR and DM rates of 28% and 34%, respectively, demonstrate that non‐operative therapy can provide acceptable local control, and may be comparable to surgery. Moreover, our observed recurrence rates directly address previously published data which favored surgical resection over DCCRT as a result of poor local control (approximately 50% failure) observed with the latter treatment, despite comparable survival observed with both approaches.^17^, ^18^ However, it is worth noting that our exclusion of patients who received esophageal surgery <6 months post‐DCCRT (done to avoid inflation of outcomes with trimodality treatment) carries a limitation in that patients who only received surgery due to failure of DCCRT may have also been excluded.

One noteworthy consideration in assessing treatment efficacy in this study is RT dose. In this cohort, a median total dose of 50.4 Gy was administered, comparable to that reported in the previously mentioned studies. Increased RT dose was not associated with any type of recurrence or a clinically meaningful impact on OS or DFS (Hazard Ratio = 0.999, *p* < 0.05). However, the role for RT dose escalation in the treatment of EC has remained an area of debate. While initial results from the INT0123 trial were largely negative,^19^ more recent data have challenged such findings. ^9^, ^20^ In a Phase I/II trial of chemoRT with simultaneous integrated boost of RT to 58.8–63.0 Gy in unresectable locally advanced EC, Chen et al. reported a median OS of 21.5 months (range, 2.3–86.4 months) and a 1‐year OS rate of 78.3%, demonstrating favorable results in a single‐arm study. In addition, LRR rates were 30% and 33% at 1 and 2 years, respectively.^20^ More recently, the ART DECO trial presented in abstract form at ASCO GI 2020 showed results that were more aligned with those from our study and INT0123. In this randomized, multi‐institutional trial, standard dose IMRT was compared to dose escalated IMRT. No difference in local control, progression free survival, or overall survival was found between the two arms, while increased toxicity was noted in the dose escalated arm, and final publication is pending.

An equally noteworthy consideration in evaluating the efficacy of DCCRT for this patient population is chemotherapy regimen. To date, no single regimen has shown definitive superiority. One of the most frequently used regimens has been combination cisplatin and fluorouracil, with median survival rates ranging from 10 to 25 months, depending on tumor histology, disease stage, concurrent RT regimen, and additional therapies received.^13^, ^18^, ^19^, ^21^, ^22^ Other fluorouracil‐based therapies such as FOLFOX or cisplatin/fluorouracil in combination with docetaxel or etoposide have also been reported, with phase 2/3 studies of mostly SCC patients reporting median OS rates of 20.2 months, 29 months, and 14.9 months, respectively.^12^, ^17^, ^23^ Platinum/taxane regimens have also been used frequently, with reported OS rates of 15–20 months, again mostly in SCC patients.^24^, ^25^ In our study cohort, MVA and stepwise regression failed to reveal a statistically significant difference in OS or DFS with carboplatin/paclitaxel or fluorouracil‐based regimens. However, AC patients had higher median OS with fluorouracil‐based regimens (24.7 vs 22.3 months) while SCC patients had higher median OS with carboplatin/paclitaxel (34.2 vs 16.4 months). In addition, fluorouracil patients were more likely to need a dose reduction or treatment, most commonly due to hematologic toxicity, suggesting decreased tolerability with this regimen. Nevertheless, as the study results reported here are retrospective and non‐randomized, comparison and direct evaluation is difficult in the setting of potential confounders. We await results of trials such as the “YYY” and “ZZZ” which may shed light on the optimum chemotherapy strategy to use for concurrent treatment.

With respect to DCCRT treatment tolerability, we observed short‐term safety and acceptable toxicity profiles when compared to surgical complications. Adverse events associated with esophageal surgery have been well‐reported in the literature, and are often limiting factors in providing optimal treatment for EC. Post‐surgical complications are the strongest risk factor for poor quality of life and delayed or incomplete recovery following treatment, with patients commonly experiencing loss of appetite, cachexia, and severe dysphagia.^26^, ^27^, ^28^ Tepper et al. reported surgical complications in 48 of 52 patients who received either surgery alone or trimodality therapy for EC, citing red blood count transfusion, postoperative fever, pneumonia requiring antibiotics, respiratory failure, wound infections, and empyema as the most common complications, and one patient dying within 30 days of surgery from surgical complications.^7^ In contrast, patients treated with DCCRT have demonstrated faster and more complete recovery than those who undergo surgery, with the more significant adverse events typically secondary to the chemotherapy component of treatment.^29^ Previous studies of DCCRT have reported severe and life‐threatening side effects occurring in 44% and 20% of patients, respectively.^13^, ^17^ In our study population, acute grade 4 toxicities occurred in 7% of patients (89% hematologic), and the only acute grade 3 toxicities occurring in >5% of patients were hematologic cytopenia (35%), dysphagia (23%), and anorexia (19%). Fluorouracil‐based therapies resulted in a higher number of dose reductions and treatment breaks compared to carboplatin/paclitaxel (48% vs 35%), however, a majority of patients (62%) were able to complete either regimen without breaks or dose omissions. In addition, while 36% of patients experienced an increase in ECOG performance status post‐RT, a majority of patients experienced no change or a decrease in ECOG performance status (51%). As such, these results not only attest to the safety and tolerability of DCCRT for EC, but also highlight the more favorable side effect profile experienced by patients undergoing DCCRT compared to surgery.

A secondary objective of this study was to identify predictors of disease‐control outcomes. One variable of particular interest was change in primary tumor SUV on PET/CT pre‐ and post‐chemoRT, which was found to be significantly associated with OS and DFS, suggesting the utility of using metabolic response in evaluating prognoses. Tumor histology was also a variable of interest, as most studies of DCCRT for non‐operable EC have focused on SCC, with very little reports of outcomes in AC. While DCCRT has traditionally been avoided for esophageal AC, our data support the use of contemporary DCCRT as a viable treatment option for patients with non‐operable disease, with a potential for durable control in a minority of patients.

Limitations to our study include the retrospective nature and non‐randomized design, which allowed for variability in chemotherapy and RT regimens used. In particular, receipt and type of induction/adjuvant therapy varied greatly, along with disease stage and follow‐up procedures. Due to the heterogeneity in follow‐up procedures, a clinical response rate was not calculated. In addition, retrospective determination of cause of death was difficult, and longer follow‐up in a greater number of patients is needed to best assess late toxicity.

## 5 CONCLUSION

The results of our study have shown that treatment of non‐operable EC with DCCRT has acceptable toxicity and can provide multi‐year disease control in some patients, even in AC. Continued follow‐up to further evaluate long‐term outcomes and investigation in large cohort studies would be useful in informing stronger conclusions about the efficacy of this treatment approach.

## CONFLICTS OF INTEREST

JRE: Bristol‐Myers Squibb (spouse employment), Novartis, Exelixis, Lexicon (consulting/advisory relationship), Calithera Biosciences, Leap Therapeutics, Merck, Bristol‐Myers Squibb, EMD Serano, Symphogen, Medimmune, Bayer, Placon Therapeutics (research funding). TBK: Celgene, Stand Up to Cancer (SU2C), Sirtex (grants), Syndax Corporation, Eli Lilly, Bristol‐Myers Squibb Taiho, H3Biomedicine (grants and nonfinancial support). JMM: Varian Medical System (advisory board, personal fee from, grant funding), Ion Beam Applications, Provisions (advisory board). JPP: Serves on committees for American Board of Radiology, American Society for Radiation Oncology, International Lymphoma Radiation Oncology Group. The other authors have no conflicts of interest to declare.

## AUTHOR CONTRIBUTIONS

All of the authors have made substantial contributions to conception and design, acquisition of data, and analysis and interpretation of data. All have been involved in drafting the manuscript or revising it critically for important intellectual content and have given final approval of the version to be published.

## Supporting information

Table S1‐2

Table S1‐2_1

## Data Availability

The data that support the findings of this study are available from the corresponding author upon reasonable request.
