# Supplementary material for: The efficacy and safety of definitive concurrent chemoradiotherapy for non‐operable esophageal cancer
Source: Cancer Med. 2021 Jan 20;10(4):1275–88. doi: 10.1002/cam4.3724 (PMC7926027; doi:10.1002/cam4.3724)
Supplement: Supplementary file 1 — Table S1‐2 [file CAM4-10-1275-s001.docx]

**SUPPLEMENTARY MATERIALS**

**Supplementary Table S1.** Number of maximum late grade 1-3 toxicities per radiation oncology follow-up (%)^[[1]](#footnote-1)^

|  | Carboplatin/paclitaxel (n=25) and Fluorouracil-based (n=5) | | | |
| --- | --- | --- | --- | --- |
| Toxicity Grade | 1 | 2 | 3 | Total |
| Upper aerodigestive & respiratory  Cough  Dyspnea  Mucositis  Dysphagia  Esophageal pain  Esophagitis  Hoarseness  Radiation pneumonitis | 7 (23)  5 (17)  1 (3)  7 (23)  3 (10)  1 (3)  3 (10)  3 (10) | 0  1 (3)  0  4 13)  1 (3)  1 (3)  0  0 | 0  0  0  3 (10)  0  0  0  0 | 7 (23)  6 (20)  1 (3)  14 (47)  4 (13)  0  0  0 |
| Gastrointestinal  Anorexia  Dehydration  Nausea/vomiting | 4 (13)  2 (7)  5 (17) | 3 (10)  0  0 | 2 (7)  0  0 | 9 (30)  2 (7)  5 (17) |
| Skin  Radiation dermatitis | 2 (7) | 0 | 0 | 2 (7) |
| Other  Fatigue  Depression | 12 (40)  4 (13) | 5 (17)  1 (3) | 0 (0)  0 (0) | 17 (57)  5 (17) |

**Supplementary Table S2.** Relationships between patient, disease, or treatment characteristic and incidence of locoregional recurrence, distant metastasis, or overall recurrence

|  | LRR | P-value | DM | P-value | LRR/DM | P-value |
| --- | --- | --- | --- | --- | --- | --- |
| **Patient Characteristic** | | | | | | |
| **Age (yr.)** | | | | | | |
| <60 | 8 (35%) | 0.112 | 10 (43%) | **0.023** | 15 (65%) | **0.007** |
| 60-70 | 10 (33%) |  | 14 (47%) |  | 16 (53%) |  |
| 70-80 | 14 (27%) |  | 15 (29%) |  | 23 (44%) |  |
| >80 | 4 (16%) |  | 5 (20%) |  | 7 (25%) |  |
| **Sex** | | | | | | |
| Female | 1 (3%) | **<0.001** | 7 (22%) | 0.132 | 7 (32%) | **0.001** |
| Male | 35 (36%) |  | 37 (38%) |  | 54 (55%) |  |
| **Smoker** | | | | | | |
| No | 9 (36%) | 0.326 | 11 (44%) | 0.247 | 13 (52%) | 0.658 |
| Yes | 27 (35%) |  | 33 (31%) |  | 48 (46%) |  |
| **Barrett’s esophagus** | | | | | | |
| No | 24 (24%) | 0.097 | 34 (34%) | 1.000 | 44 (44%) | 0.205 |
| Yes | 12 (41%) |  | 10 (34%) |  | 17 (59%) |  |
| **PPI use pre-diagnosis** | | | | | | |
| No | 20 (30%) | 0.559 | 27 (41%) | 0.097 | 33 (50%) | 0.488 |
| Yes | 16 (25%) |  | 17 (27%) |  | 28 (44%) |  |
| **Body weight loss, last 6 mo.** | | | | | | |
| $\leq$10% | 25 (30%) | 0.419 | 26 (31.7%) | 0.566 | 39 (48%) | 0.858 |
| >10% | 11 (23%) |  | 18 (37.5%) |  | 22 (46%) |  |
| **Disease Characteristic** | | | | | | |
| **Tumor histology^a^** | | | | | | |
| AC | 24 (30%) | 0.549 | 28 (35%) | 0.850 | 40 (50%) | 0.471 |
| SCC | 12 (24%) |  | 16 (33%) |  | 21 (43%) |  |
| **Tumor site, esophagus^a^** | | | | | | |
| Upper | 2 (29%) | 1.000 | 3 (43%) | 0.652 | 4 (57%) | 0.848 |
| Middle | 6 (25%) |  | 10 (42%) |  | 12 (50%) |  |
| Lower, GEJ | 22 (27%) |  | 27 )33%) |  | 38 (47%) |  |

**Supplementary Table S2.** Continued

| **Clinical tumor stage^a^** | | | | | | |
| --- | --- | --- | --- | --- | --- | --- |
| T1 | 0 (0%) | 0.272 | 2 (33%) | 0.410 | 2 (33%) | 0.361 |
| T2 | 7 (29%) |  | 7 (29%) |  | 10 (42%) |  |
| T3 | 23 (29%) |  | 30 (38%) |  | 42 (53%) |  |
| T4 | 3 (43%) |  | 3 (43%) |  | 3 (43%) |  |
| **Clinical nodal stage^a^** | | | | | | |
| N0 | 7 (15%) | **0.013** | 13 (27%) | 0.129 | 17 (35%) | **0.042** |
| N+ | 22 (36%) |  | 31 (41%) |  | 42 (55%) |  |
| **Treatment Characteristic** | | | | | | |
| **Feeding tube pre-chemoRT** | | | | | | |
| No | 21 (32.3%) | 0.327 | 17 (26.2%) | 0.095 | 30 (46%) | 1.000 |
| Yes | 15 (23.1%) |  | 27 (41.5%) |  | 31 (47.7%) |  |
| **Feeding tube post-chemoRT** | | | | | | |
| No | 19 (32.2%) | 0.329 | 17 (28.8%) | 0.352 | 28 (47%) | 1.000 |
| Yes | 17 (23.9%) |  | 27 (38.0%) |  | 33 (46%) |  |
| **Chemotherapy regimen^a^** | | | | | | |
| Carboplatin/paclitaxel | 24 (24%) | 0.166 | 35 (35%) | 0.664 | 44 (44%) | 0.410 |
| Fluorouracil-based | 12 (39%) |  | 7 (24%) |  | 15 (52%) |  |
| **RT dose (Gy)** | | | | | | |
| $\leq$50.4 | 16 (25%) | 0.693 | 19 (30%) | 0.456 | 28 (43%) | 0.598 |
| >50.4 | 19 (29%) |  | 24 (37%) |  | 32 (49%) |  |
| **RT modality** | | | | | | |
| 3DCRT | 6 (30%) | 0.791 | 7 (35%) | 1.000 | 8 (40%) | 0.628 |
| IMRT/PBT | 30 (27%) |  | 37 (34%) |  | 53 (48%) |  |
| **Change in primary tumor PET SUV^[[2]](#footnote-2)^** | | | | | | |
| <50% | 8 (36%) | 1.000 | 12 (54%) | 0.613 | 15 (68%) | 0.793 |

1. No grade 4-5 toxicities [↑](#footnote-ref-1)
2. Abbreviations: LRR, locoregional recurrence; DM, distant metastasis; PPI, proton pump inhibitor; AC, adenocarcinoma; SCC, squamous cell carcinoma; GEJ, gastroesophageal function; chemoRT, chemoradiation; RT, radiation therapy; 3DCRT, 3-D conformal radiation therapy; IMRT, intensity modulated radiation therapy; RT, radiation therapy; PBT, proton beam therapy; PET/CT SUVmax, positron emission tomography / computed tomography maximum standardized uptake value

   Unknowns / other omitted for brevity [↑](#footnote-ref-2)
